# Adipocyte Ceramides—The Nexus of Inflammation and Metabolic Disease

**DOI:** 10.3389/fimmu.2020.576347

**Published:** 2020-09-23

**Authors:** Bhagirath Chaurasia, Chad Lamar Talbot, Scott A. Summers

**Affiliations:** ^1^Division of Endocrinology, Department of Internal Medicine, Carver College of Medicine and the Fraternal Order of Eagles Diabetes Research Center, University of Iowa, Iowa City, IA, United States; ^2^Department of Nutrition and Integrative Physiology and the Diabetes and Metabolism Research Center, University of Utah, Salt Lake City, UT, United States

**Keywords:** ceramide, inflammation, insulin, diabetes, adipocyte

## Abstract

Adipose depots are heterogeneous tissues that store and sense fuel levels. Through the secretion of lipids, cytokines, and protein hormones (adipokines), they communicate with other organ systems, informing them of the organism's nutritional status. The adipose tissues include diverse types of adipocytes (white, beige, and brown) distinguished by the number/size of lipid droplets, mitochondrial density, and thermogenic capacity. Moreover, they include a spectrum of immune cells that modulate metabolic activity and tissue remodeling. The unique characteristics and interplay of these cells control the production of ceramides, a class of nutrient signals derived from fat and protein metabolism that modulate adipocyte function to regulate glucose and lipid metabolism. The excessive accumulation of ceramides contributes to the adipose tissue inflammation and dysfunction that underlies cardiometabolic disease. Herein we review findings on this important class of lipid species and discuss their role at the convergence point that links overnutrition/inflammation to key features of the metabolic syndrome.

## Introduction

Obesity increases one's risk for metabolic diseases such as diabetes, coronary artery disease, non-alcoholic steatohepatitis, and heart failure. The condition promotes (a) the accumulation of deleterious lipid metabolites in non-adipose tissues (i.e., lipotoxicity) and (b) chronic low-grade inflammation, which in turn produces the tissue dysfunction that fuels these disorders. The lipotoxicity is secondary to adipose dysfunction, such that excessive lipids are delivered to peripheral tissues rather than being safely stored as triglycerides within the healthy adipocyte (1–5). The inflammation results from the increased recruitment of pro-inflammatory macrophages into the expanded adipose depots, leading to increased secretion of inflammatory cytokines such as tumor necrosis factor-α (TNF-α), interleukins (IL), and chemokines (6–8). Together, these lipotoxic and inflammatory pathways account for virtually all of the features of the metabolic syndrome including insulin resistance, dyslipidemia, and hypertension.

Lipids, in addition to being major fuel reservoirs (e.g., triglycerides), have important roles in the regulation of nutrient storage. In particular, sphingolipids such as ceramides are metabolic signals that accumulate in obesity and trigger evolutionarily conserved cellular responses to lipid overload (9). Such mechanisms include inhibiting the uptake of glucose and amino acids, leading to the preferential utilization of free fatty acids (FFAs) for energy; slowing rates of triglyceride lipolysis; and impairing mitochondrial respiration (9). At higher concentrations, ceramides induce apoptosis (9). These sphingolipid actions contribute to the tissue dysfunction that underlies non-alcoholic steatohepatitis, diabetes, and heart disease. Inflammatory cytokines, including TNF-α and IL-1, reinforce this signal by accelerating ceramide production (10). Ceramides thus function at the nexus of lipid metabolism and inflammation.

Studies in mice reveal that inhibition of ceramide synthesis resolves hepatic steatosis and improves insulin-stimulated glucose disposal to slow the progression of cardiometabolic diseases (11). These ceramide-lowering interventions also alter adipose tissue metabolism and morphology, enhancing glucose utilization, and energy expenditure. These manipulations also decrease adipose tissue inflammation and alter macrophage polarization, converting them from pro-inflammatory M1-macrophages into anti-inflammatory M2-macrophages (12). Herein we will review the synergy between the free fatty acids (FFAs) and ceramides that accumulate in obesity and inflammation that accompanies adipose tissue expansion for the development of cardiometabolic diseases. In addition, we will discuss the potential therapeutic approaches for targeting ceramides to reduce inflammation and improve adipose health.

## Excess Free Fatty Acids Induce Metabolic Disorders

Elevations in circulating FFA resulting from increased nutrient consumption or unchecked lipolysis have been implicated in metabolic disorders including insulin resistance, type 2 diabetes, and cardiovascular disease (13). Emerging studies suggest that these fatty acids fuel production of deleterious lipid metabolites such as ceramides while inducing chronic inflammation (3, 6, 14, 15). To this end, FFA, particularly saturated fatty acids such as palmitate which is a key substrate for ceramide production while also modulating innate immune cells to elicit a proinflammatory response, have important roles at the origin of metabolic disease (16, 17).

## Pathways Controlling Ceramide Synthesis and Metabolism

**Figure 1:**
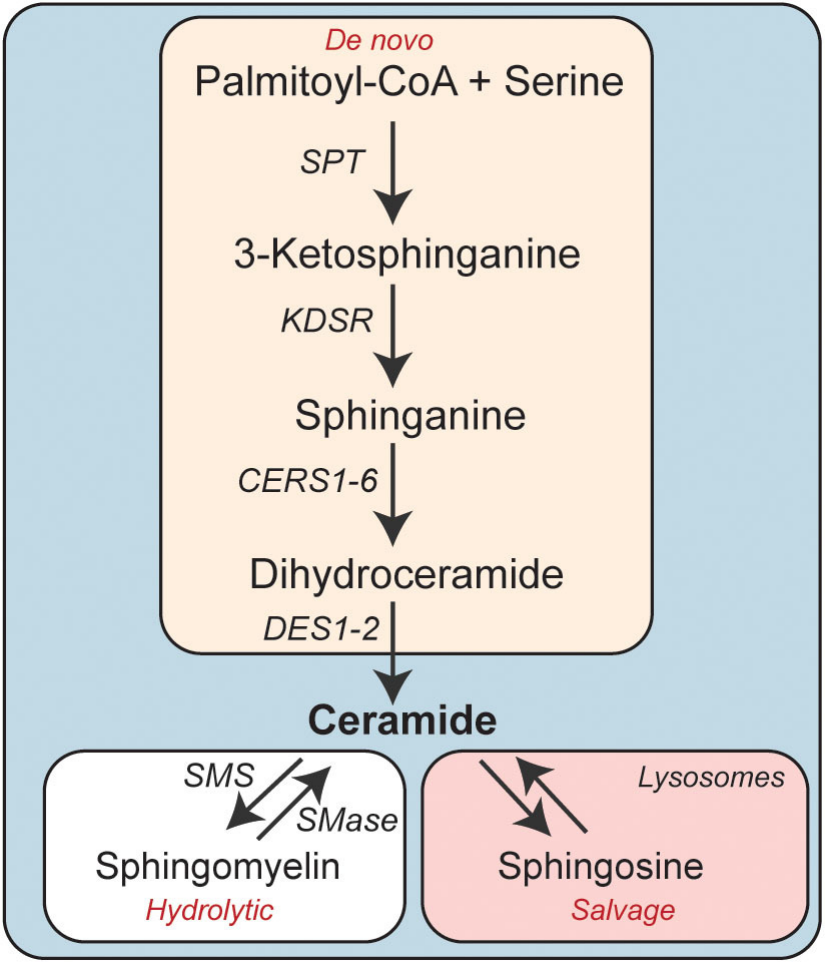
Schematic depicting the enzymatic pathways involved in cellular ceramide synthesis. CERS, Ceramide synthase; DES, Dihydroceramide desaturase; KDSR, 3-ketodihydrosphinganine reductase; SMS, Sphingomyelin synthase; SMase, Sphingomyelinase.

Ceramides are precursors of complex sphingolipids (e.g., sphingomyelin) that are integral components of cell membranes. The sphingolipid content of the adipose depots is influenced by nutrient availability (e.g., increased levels of sphingolipid precursors such as serine and palmitate), inflammatory signals, adiponectin, and other factors that control global stress responses. Ceramides can thus serve as metabolic messengers that integrate input from a variety of factors associated with obesity and metabolic disease. Their cellular levels are determined by three enzymatic pathways: *de novo* synthesis, sphingomyelin hydrolysis, and the salvage pathway (Figure 1) (18, 19).

The *de novo* synthesis pathway comprises four sequential enzymatic steps (20). Serine palmitoyltransferase (SPT) catalyzes the first reaction, condensing palmitoyl-CoA (CoA) and serine to produce 3-ketosphinganine. This transient intermediate doesn't accumulate in cells, as it is rapidly converted to sphinganine by 3-ketosphinganine reductase (*3Ksn*). Ceramide synthases (CERS1-6) then add a fatty acid, ranging in chain length from 14-carbon to 34-carbon atoms, to sphinganine to produce dihydroceramides. The CERS enzymes have variable substrate specificity and unique tissue distributions and account for much of the diversity in sphingolipids (21). In the fourth and final step, dihydroceramide desaturase (*Degs1* and *2*) introduces a critical double-bond into dihydroceramide, generating ceramides (22).

The second pathway involves the hydrolysis of sphingomyelin by neutral or acid sphingomyelinase to produce phosphocholine and re-form ceramide (23).

The third pathway, termed the salvage pathway, allows for the reformation of ceramides from sphingolipids after they are degraded in late endosomes or lysosomes (24). The liberated sphingoid base can be re-acylated by the aforementioned CERS enzymes, re-synthesizing ceramides.

## Plasma and Adipose Ceramides Correlate With Free Fatty Acids, Markers of Inflammation, and the Severity of Cardiometabolic Diseases

Within the last decade, advances in mass-spectrometry have allowed researchers to confidently assess whether plasma and tissue ceramide levels correlate with indices of metabolic diseases. Numerous groups have found that circulating ceramides and FFAs are elevated in subjects with insulin resistance, type 2-diabetes, non-alcoholic fatty liver diseases, chronic kidney diseases and major adverse cardiovascular events including mortality (25–35). In parallel, researchers have shown that circulating inflammatory cytokines also positively associate with these metabolic outcomes (36, 37). Interestingly, circulating FFAs, ceramides and inflammatory cytokines also correlate with one another in human subjects with coronary artery disease, hepatic steatosis, or insulin resistance (10, 33, 38–40). These studies suggest that they may have interrelated roles in the etiology of metabolic disorders.

Adipose tissue ceramide content and inflammation have also been evaluated in subjects with obesity, insulin resistance and/or diabetes. One such study, by the Yki-Jävinen group, demonstrated that ceramide levels are elevated in the adipose tissues of individuals with insulin resistance, independent of obesity (41). In this study, the tissue also showed increases in inflammatory markers. The Brüning laboratory also found that ceramides, particularly C_16_-ceramides, were elevated in individuals with obesity (42). They also observed dramatically increased expression of ceramide synthase 6 (CERS6), the enzyme that is responsible for generating C_16_-ceramides. Additionally, CERS6 expression positively correlated with insulin resistance.

## Ceramides Are Modulated by Several Inflammatory and Anti-Inflammatory Signaling Molecules

The oversupply of precursors such as palmitate and serine undoubtedly account for much of the ceramide accumulation that occurs in obesity. Indeed, a small number of dietary studies have shown that dietary fat intake influences ceramide synthesis and accumulation (43, 44). As outlined below, inflammatory modulators also influence the rate of ceramide production.

### Tumor Necrosis Factor-Alpha (TNF-α) Produces Ceramides to Contribute to Insulin Resistance

In obesity, the recruitment of macrophages to the expanding adipose depots can induce an inflammatory state characterized by increased expression and secretion of inflammatory cytokines such as TNF-α, IL-6, and IL-1β (6, 15, 45–50). Some of these cytokines have been shown to produce ceramides (51–53). In particular, serum and adipose TNF-α are often elevated in individuals with obesity and/or type 2 diabetes and correlate with the severity of insulin resistance (54–56) and with levels of ceramides (33). In cultured cells, the cytokine stimulates ceramide accumulation by inducing expression of ceramide synthesis genes [e.g., serine palmitoyltransferase (SPT)] and increasing expression and activity of sphingomyelin hydrolyzing enzymes (e.g., sphingomyelinase) (51, 57–62). Similar effects on ceramide synthesis have been demonstrated with certain cytokines such as the TNF-α *in vivo* (63), which antagonize insulin-stimulated glucose disposal in rats and humans (64, 65). In cultured adipocytes and myeloid cells, researchers confirmed that it inhibits insulin signaling and action via receptor-mediated activation of sphingomyelinase (66).

In mice, genetic manipulations to ablate TNF-α or its receptors ameliorate obesity-induced insulin resistance (46, 67). However, clinical trials targeting TNF-α have generally shown little or no beneficial effect on systemic insulin sensitivity (68, 69), indicating that TNF-α lowering is insufficient to combat insulin resistance in humans.

### Toll-Like Receptors Induce Ceramide Biosynthesis to Contribute to Insulin Resistance

The lipotoxic environment in obesity increases the supply of saturated fatty acids that either directly or indirectly activate toll-like receptor (TLR)-4 (70–74). These pattern recognition receptors, which are typically involved in innate immune responses, have been implicated in inflammation and insulin resistance that accompanies obesity and underlies metabolic disease. For example, Flier et al. found that mice lacking TLR-4 were protected from lipid or high fat diet-induced insulin resistance (17, 75). They also found that long-chain fatty acids signal via TLR-4 to induce transcription of inflammatory cytokines (e.g., TNF-α and IL-6), thus reinforcing and enhancing the inflammatory state. Using similar approaches with various loss-of-function TLR-4 mouse models, four other laboratories described essential roles for TLR-4 in obesity and/or insulin resistance (76–79). Curiously, Shulman et al. found the opposite result, concluding that TLR-4 was not required for lipid-induced insulin resistance (80).

Activation of toll-like receptor (TLR)-4, via lipopolysaccharides (LPS) or a more specific ligand Kdo(2)-lipid A, induces ceramide accumulation by increasing the expression of several ceramide synthesis enzymes (77, 81–84). In cultured myotubes, nuclear factor kappa B (NFκB) was found to be an obligate intermediate in these TLR-4 mediated effects on ceramide production (77). In contrast, ablation of TLR-4 in mice reduces ceramides, and even prevents their synthesis in models of lipid oversupply (i.e., mice fed a high fat diet or infused with lipid cocktails) (77). These findings indicate that TLR-4 enhances ceramide production and reveal the interplay between TLR-4 and ceramides in the metabolic dysfunction that accompanies obesity.

The mechanisms controlling TLR-4 activation in obesity have been controversial. Though saturated fatty acids were initially speculated to be TLR-4 ligands (70–73), some have argued that fatty acids signal through indirect signaling mechanisms (74). Others have argued that this observation is an artifact, likely due to contamination of the saturated fatty acid preparations with lipopolysaccharide (85). In an elegant study, Lancaster et al. found that saturated fatty acids do not bind directly to the TLR-4 receptors, but rather prime TLR-4 to induce lipid-mediated inflammatory signaling (74). These authors found that activating TLR-4 led to a marked upregulation of ceramides and ceramide-synthesizing genes (74).

### Ceramides Activate the NLRP3 Inflammasome to Increase Cytokine Secretion

Inflammasomes are large, multiprotein complexes that form in response to endogenous stress signals, initiating a wide range of cellular activities that include production of the pro-inflammatory cytokines (e.g., IL-1β). The best characterized inflammasome is termed NLRP3 because of the presence of NOD-, LRR-, and pyrin domain-containing protein 3 within the complex. Other components include the adapter ASC and pro-caspase-1. Saturated FFAs were recently found to induce inflammasome activation in macrophages, prompting speculation that lipotoxic intermediates such as ceramides might drive inflammasome activation (49). In both macrophages and adipocytes, ceramides activate the NLRP3 inflammasome, promoting cleavage of caspase-1 and subsequent stimulation of cytokine secretion (86). Subsequent studies found roles for inflammasomes as a downstream ceramide effector in other cell types (87–89), Within adipocytes, this ceramide interaction with the NLRP3 inflammasome may contribute to the adipose inflammation that contributes to insulin resistance. Interestingly, inhibiting *de novo* ceramide biosynthesis in macrophages did not influence the inflammasome (90), nor did it impact glucose tolerance (11, 12, 90). Moreover, palmitate has been shown to elicit activation of inflammasome by modulating the AMPK-ROS-autophagy pathway, suggesting alternative mechanisms link FFAs to this immune complex (49).

### Plasminogen Activator Inhibitor-1 Has a Bidirectional Relationship With Ceramides

Plasminogen activator inhibitor-1 (PAI-1) is a glycoprotein that is synthesized in endothelial cells, liver, adipose tissue, and other tissue types. It inhibits the serine proteases that covert plasminogen into the active fibrinolytic enzyme plasmin (91, 92). Plasma PAI-1 concentrations are elevated in obesity and diabetes and correlate with the severity of insulin resistance (93–95). Pharmacological inhibition or genetic ablation of PAI-1 in mice protects them from both obesity and insulin resistance while improving adipocyte health and decreasing adipose inflammation (96–99). PAI-1 ablation ensures this protection, at least in part, by reducing accumulation of ceramides in adipocytes, which it accomplishes by decreasing expression of ceramide synthesis genes (96). Conversely, ceramides were reported to induce PAI-1 expression in adipocytes (100), revealing bidirectional interplay between PAI-1 and ceramides that modulates adipose tissue inflammation and function.

### Adiponectin Receptors Are Ligand Activated Ceramidases

The adipokine adiponectin attenuates many features of diabetes and heart disease, including insulin resistance, dyslipidemia, inflammation and cardiomyocyte, endothelial cell and beta-cell apoptosis (101–106). Holland, Scherer et al. were intrigued by the fact that adiponectin and ceramides have such oppositional roles in biology. Moreover, they observed a sequence similarity between adiponectin receptors (AdipoRs) and a family of ceramidases. They thus tested the provocative idea that adiponectin elicited its broad spectrum of actions by reducing (via diacylation) ceramides. They confirmed that the receptor had ceramidase activity that is activated by ligand binding (105). In mice, the cardioprotective and anti-diabetic actions of adiponectin were accompanied by reductions in ceramides (105). Moreover, they identified key residues in AdipoRs that were required for ceramidase activity and for all of adiponectin's downstream actions (105). These findings were then validated by Vasiliauskaite-Brooks et al. who crystalized the AdipoRs in presence of short-chain ceramide analogs, discovering it bound to the liberated sphingoid base (107, 108). They also confirmed that the purified receptors possess ceramidase activity (107, 108). These studies suggest yet another key regulatory mechanism that controls cellular ceramides in order to modulate inflammation and other features of the metabolic syndrome.

## Convergence of Adipose Ceramides and Inflammation to Control Insulin Resistance

Insulin resistance is a defining attribute of the metabolic syndrome that increases one's risk for diabetes and heart disease. As noted above, numerous studies have described correlational relationships between insulin resistance, circulating cytokines, and ceramides in clinical populations (9, 21, 22). Studies in rodents further indicate that ceramides play causative roles in insulin resistance, often linking inflammatory agonists to their deleterious effects on glucose uptake and utilization.

**Figure 2:**
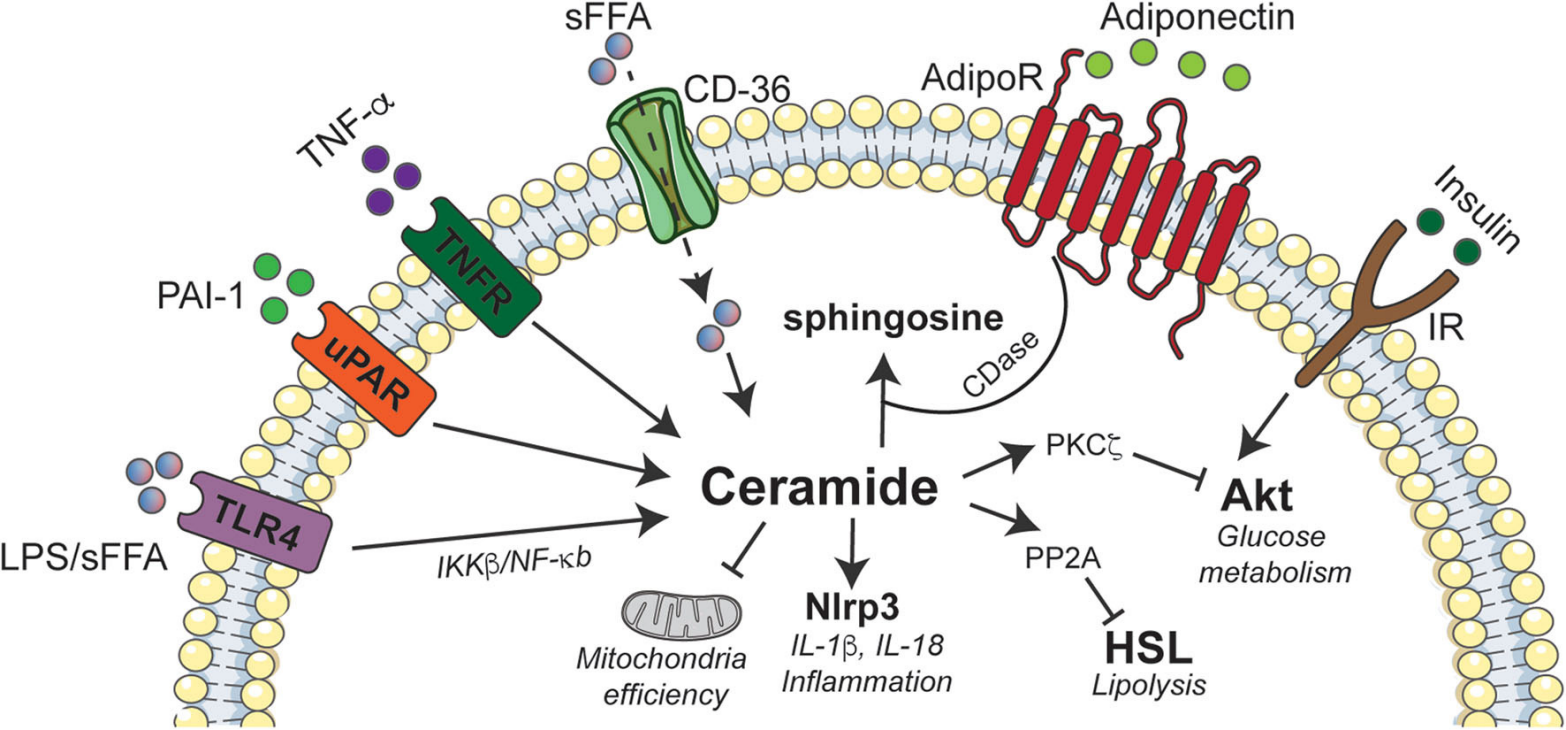
Schematic depicting interactions between ceramides and inflammatory agonists in adipose tissue. Ceramide accumulation elicits deleterious effects on adipose tissue function by activating Nlrp3 inflammasome that induces inflammation, inhibition of Akt via PKCζ to abrogate insulin signaling, and promoting excessive lipid storage by inhibiting HSL. The immunomodulatory adiponectin exhibits some of its beneficial effects by stimulating ceramidase activity that converts ceramides to sphingosine. Akt, Protein Kinase B; CD-36, cluster of differentiation 36; AdipoR, Adiponectin receptor; CDase, Ceramidase; IKK, Ikappa kinase; IL, interleukin; IR, insulin receptor; LPS, lipopolysaccharide; NF-κB, Nuclear factor kappa-light-chain-enhancer of activated B cells; Nlrp3, NLR family, pyrin domain containing 3; PAI-1, Plasminogen activator inhibitor 1; PKC, protein kinase C; PP2A, Protein phosphatase 2A; sFFA, Saturated fatty acids; TLR4, Toll like receptor-4; TNF-α, Tumor necrosis factor alpha; TNFR, Tumor necrosis factor alpha receptor; uPAR, Urokinase-type plasminogen activator receptor.

The earliest studies evaluating the role of ceramides in insulin resistance analyzed their effects in 3T3-L1 adipocytes, a murine cell line that shows many of the hallmark metabolic attributes of human adipose tissue. Those studies revealed that ceramides inhibit glucose uptake by inhibiting activation of Akt/PKB (109), a serine/threonine kinase that is an obligate intermediate in insulin-stimulated glucose transporter GLUT4 translocation, as well as glycogen and protein synthesis and protection from apoptosis. Curiously, ceramides did not inhibit the signaling events that precede Akt/PKB activation, such as the activation of PI3-kinase or generation of its product, 3'-polyphosphoinositides (110). Moreover, they blocked activation of the enzyme by numerous other stimuli, including those that don't utilize the signaling scaffold insulin receptor substrate-1 (110), which had recently been identified as a putative site of insulin resistance (111). This observation prompted a flurry of studies seeking to elucidate the signaling mechanisms that linked elevations in ceramides to the inhibition of this important enzyme. These studies revealed that ceramides inhibit Akt/PKB by two known mechanisms, which impact different portions of the enzyme (112). Ceramides dephosphorylate key activating residues through protein phosphatase 2A(PP2A) (112), which is an established ceramide effector (113). Through an alternate mechanism, ceramide blocks the translocation of Akt/PKB to the plasma membrane (112). Studies by the Hundal laboratory subsequently revealed that the translocation effect was due to ceramide actions on atypical protein kinase C (PKCζ), which phosphorylates a key residue in the pleckstrin homology domain of Akt/PKB to block its recruitment to the plasma membrane (114–117). These disparate ceramide mechanisms are clearly separable, as they impact different protein domains and are responsive to distinct inhibitors (112). They also vary by cell type, seeming to be contingent on the relative quantity of caveolar membranes. Adipocytes that have a high abundance of caveolae favor the PKCζ-Akt/PKB axis rather than the PP2A-Akt/PKB axis (118) (Figure 2).

These studies suggested that ceramides, induced by either the oversupply of fatty acid substrates or the inflammation-induced upregulation or activation of ceramide-producing enzymes, might drive insulin resistance *in vivo*. Data in rodents support this hypothesis. For example, a pharmacological inhibitor of SPT (i.e., myriocin) prevents and/or reverses insulin resistance in high fat diet fed mice (12, 77, 119–121), lipid-infused rats (121), fructose-fed hamsters (122), and leptin-deficient mice and rats (i.e., Zucker *fa/fa* rats and *ob/ob* mice) (121). It also resolves steatosis, decreases adipocyte size, and enhances recruitment of M2 macrophages into subcutaneous adipose tissue (12). Similar findings were obtained with pharmacological (i.e., fenretinide) or genetic (i.e., gene knockout) inhibition of DES1 (11, 123, 124). Many of these actions could be explained by ceramide actions within the adipocyte. Adipocyte-specific depletion of SPTLC2, a critical subunit within the SPT complex, or DES1 improved insulin sensitivity, resolved hepatic steatosis, and decreased inflammation of the adipose beds (12). A comparable spectrum of effects was obtained using adipose-specific over-expression of acid ceramidase (125).

While the mechanisms that allow ceramide to modulate lipid and inflammation-induced insulin resistance are fairly clear, the means by which adipocyte ceramides induce the recruitment of macrophages are not. Of note, most of the protective actions of ceramide depletion are unlikely to be driven by ceramides within the macrophage, as depleting SPTLC2 or DES1 from myeloid cells did not influence glucose homeostasis (11, 12, 90).

## Convergence of Adipose Tissue Ceramides and Inflammation to Control Energy Expenditure

In mice, myriocin also increases energy expenditure via a mechanism that involves changes to the adipose depot. The SPT inhibitor increased the allotment of adipocytes that express uncoupling protein 1 (UCP1) (12), a mitochondrial protein that dissipates the proton gradient generated by the electron transport chain. This uncoupling reduces mitochondrial membrane potential and leads to high rates of substrate oxidation, heat production and energy expenditure (126). Similar observations were obtained following the adipocyte-specific depletion of the SPTLC2 subunit (12).

Myriocin also caused a shift in macrophage polarization from M1 to M2, which has been shown to induce adipose “browning” characterized by the upregulation of UCP1. Given these data, we profiled macrophage content in adipose tissue following a myriocin intervention. This revealed a recruitment of M2-macrophages in the adipose tissue that was associated with a reduction in expression of key pro-inflammatory cytokines (e.g., IL-6, MCP-1, and TNF-α) and an induction of a crucial anti-inflammatory cytokine IL-10 (11). To resolve whether these improvements were due to cell-autonomous ceramide actions within the adipocytes or macrophages, we depleted the *Sptlc2* gene from both adipocytes and macrophages. Adipocyte-specific depletion recapitulated the effects of myriocin and increased the recruitment of M2-macrophages and expression of thermogenic genes (e.g., *Ucp1, Pgc1a*, and *Prdm16*). These data indicated that adipocyte sphingolipids likely drove the cellular responses that increased energy expenditure. By comparison, depleting *Sptlc2* from macrophages failed to impact energy expenditure. Moreover, ectopic ceramides were also shown to inhibit mitochondrial respiration and block activation of hormone-sensitive lipase by β-adrenergic agonists. The effects on lipolysis were mediated by the aforementioned ceramide effector PP2A (Figure 2).

Beyond the effects on UCP1 and HSL, ceramides seem to slow energy expenditure by inhibiting mitochondrial respiration. Indeed, addition of ceramides to cells is sufficient to inhibit mitochondrial activity (12). Hammerschmidt et al. (127) elucidated one mechanism that underlies this effect, determining that ceramides bind to mitochondrial fission factor (MFF) to alter mitochondrial morphology and reduce respiratory capacity (127). This effect is specific for the C_16_-ceramides produced by CERS6 (127).

Two other studies have evaluated the effects of reducing ceramides in white adipocytes. Curiously, while these studies did find that depleting ceramides from adipose tissue influenced glucose and lipid homeostasis, neither intervention induced adipose browning. One was the aforementioned study evaluating the consequence of acid ceramidase expression, while the other was our study involving DES1 depletion (11). While these interventions affected mitochondrial respiration, they did not induce UCP1 expression. We thus conclude that the effect on UCP1 is not due to direct ceramide actions, but rather to another intermediate in the pathway. One attractive hypothesis is that the browning effects are mediated by the CERS enzymes (128, 129), which have been shown to be transcriptional repressors that move to the nucleus and regulate lipase expression following encounters with fatty acids. By comparison, we conclude that the effects on mitochondrial fission and lipolysis (i.e., HSL) are due to direct actions of the sphingolipid analogs on MFF and PP2A, respectively. The effects on mitochondrial morphology/respiration and HSL were observed in all of the interventional studies described.

## Conclusion

Inflammation has long been known to be a hallmark of obesity, owing to the recruitment of macrophages to adipose depots and the enhancement of TLR-4 signaling by saturated fatty acids. Herein we discussed how the impact of chronic inflammation on host metabolism are linked to ceramide-driven lipotoxicity. Ceramides, which are universally upregulated by inflammatory stimuli, inhibit insulin-stimulated glucose disposal and mitochondrial respiration. They thus provide a convergence point that links overnutrition/dyslipidemia and inflammation to drive many of the key features of the metabolic syndrome. Curiously, manipulating ceramides in adipose tissue also influences the inflammatory state of the organ, suggesting the existence of feedback mechanisms that involve ceramide-dependent, adipocyte autonomous signals that control the immune cell population (e.g., via the NLRP3 inflammasome). Additional research on ceramides and their inflammatory regulators thus holds great promise as a means to combat metabolic disease and improve adipose tissue health.

## Author's Note

Some of the figures presented in this manuscript were prepared using Servier Art.

## Author Contributions

All authors listed have made a substantial, direct and intellectual contribution to the work, and approved it for publication.

## Conflict of Interest

SS is founder and consultant for Centaurus Therapeutics. The remaining authors declare that the research was conducted in the absence of any commercial or financial relationships that could be construed as a potential conflict of interest.
